# The Ras guanine nucleotide exchange factor RasGRF1 promotes matrix metalloproteinase-3 production in rheumatoid arthritis synovial tissue

**DOI:** 10.1186/ar2785

**Published:** 2009-08-13

**Authors:** Joana RF Abreu, Daphne de Launay, Marjolein E Sanders, Aleksander M Grabiec, Marleen G van de Sande, Paul P Tak, Kris A Reedquist

**Affiliations:** 1 Division of Clinical Immunology and Rheumatology, Academic Medical Center, University of Amsterdam, Meibergdreef 9, 1105 AZ Amsterdam, The Netherlands

## Abstract

**Introduction:**

Fibroblast-like synoviocytes (FLS) from rheumatoid arthritis (RA) patients share many similarities with transformed cancer cells, including spontaneous production of matrix metalloproteinases (MMPs). Altered or chronic activation of proto-oncogenic Ras family GTPases is thought to contribute to inflammation and joint destruction in RA, and abrogation of Ras family signaling is therapeutic in animal models of RA. Recently, expression and post-translational modification of Ras guanine nucleotide releasing factor 1 (RasGRF1) was found to contribute to spontaneous MMP production in melanoma cancer cells. Here, we examine the potential relationship between RasGRF1 expression and MMP production in RA, reactive arthritis, and inflammatory osteoarthritis synovial tissue and FLS.

**Methods:**

Expression of RasGRF1, MMP-1, MMP-3, and IL-6 was detected in synovial tissue by immunohistochemistry and stained sections were evaluated by digital image analysis. Expression of RasGRF1 in FLS and synovial tissue was also assessed by immunoblotting. Double staining was performed to detect proteins in specific cell populations, and cells producing MMP-1 and MMP-3. RasGRF1 expression was manipulated in RA FLS by cDNA transfection and gene silencing, and effects on MMP-1, TIMP-1, MMP-3, IL-6, and IL-8 production measured by ELISA.

**Results:**

Expression of RasGRF1 was significantly enhanced in RA synovial tissue, and detected in FLS and synovial macrophages *in situ*. In cultured FLS and synovial biopsies, RasGRF1 was detected by immunoblotting as a truncated fragment lacking its negative regulatory domain. Production of MMP-1 and MMP-3 in RA but not non-RA synovial tissue positively correlated with expression of RasGRF1 and co-localized in cells expressing RasGRF1. RasGRF1 overexpression in FLS induced production of MMP-3, and RasGRF1 silencing inhibited spontaneous MMP-3 production.

**Conclusions:**

Enhanced expression and post-translational modification of RasGRF1 contributes to MMP-3 production in RA synovial tissue and the semi-transformed phenotype of RA FLS.

## Introduction

Inflammation of affected joints in rheumatoid arthritis (RA) is characterized by infiltration of the synovial sublining by macrophages, lymphocytes, and other immune cells, and by intimal lining layer hyperplasia due to increased numbers of intimal macrophages and fibroblast-like synoviocytes (FLS) [1]. Initial *in situ *and *in vitro *studies of invasive RA FLS revealed striking similarities with transformed cells expressing mutated proto-oncogene and tumor suppressor gene products [2]. Hyperplastic FLS invading the joints of RA patients resemble proliferating tumor cells, and RA FLS proliferate more rapidly *in vitro *than FLS from inflammatory non-RA patients or healthy individuals [3]. Characteristic of transformed cells, RA FLS spontaneously secrete autocrines and matrix metalloproteinases (MMPs), display anchorage-independent growth, and are resistant to contact inhibition of proliferation [4,5]. While transforming mutations in gene products involved in cellular transformation, such as Ras and PTEN, have not been detected in RA FLS [6,7], it is appreciated that signaling pathways regulated by proto-oncogene and tumor suppressor gene products are constitutively activated due to stimulation by inflammatory cytokines, chemokines, growth factors, and oxidative stress in RA synovial tissue [8].

Ras superfamily small GTPases are expressed throughout mammalian tissue, and play essential roles in coupling extracellular stimuli to multiple downstream signaling pathways [9]. Cellular stimulation results in the activation of guanine nucleotide exchange factors (GEFs), which catalyze the exchange of GDP on inactive GTPase for GTP. The binding of GTP to Ras superfamily GTPases leads to a conformational change in the GTPase, allowing signaling to downstream effector proteins [10]. Of these small GTPases, Ras family homologs (H-Ras, K-Ras, and N-Ras) are important in coupling extracellular stimuli to activation of a shared set of signaling pathways regulating cell proliferation and survival, including mitogen-activated protein kinase cascades, phosphoinositide 3-kinase and Ral GTPases [9,11]. The related but distinct family of Rho GTPases (including Rac, Cdc42 and Rho proteins) regulate cellular polarization and chemotactic responses, mitogen-activated protein kinase cascades, and oxidative burst machinery [12,13]. GEF selectivity in activating different Ras homologs, as well as differential coupling of GEFs to specific types of cellular receptors – such as Son-of-sevenless coupling to tyrosine kinase-dependent receptors, and Ras guanine nucleotide-releasing factor 1 (RasGRF) coupling to G protein-coupled receptors – achieve specificity in Ras superfamily GTPase signaling.

Previous studies have demonstrated that Ras family homologs are present in RA synovial tissue, and are preferentially expressed in the intimal lining layer [14,15]. Activation of Ras effector pathways, including mitogen-activated protein kinases, phosphoinositide 3-kinase, and NF-κB, is enhanced in RA patients compared with disease control individuals [16-18]. In RA synovial fluid T cells, constitutive activation of Ras, in conjunction with inactivation of the related GTPase Rap1, contributes to persistent reactive oxygen species production by these cells [19,20]. In RA FLS, ectopic expression of dominant-negative H-Ras suppresses IL-1-induced extracellular signal-regulated kinase activation and IL-6 production [21]. Dominant-negative Raf kinase, which broadly binds to and inhibits Ras family members and related GTPases, suppresses epidermal growth factor-induced extracellular signal-regulated kinase and c-jun N-terminal kinase (JNK) activation in RA FLS, and reduces constitutive expression of MMPs [22]. Additionally, strategies that broadly inhibit Ras family function *in vivo *are protective in animal models of arthritis [21-23].

Evidence is now emerging that altered expression of Ras GEFs may contribute to autoimmune diseases. Mice lacking expression of the Ras GEF Ras guanine nucleotide-releasing protein 1 develop a spontaneous systemic lupus erythematosus-like disease, and similar defects are observed in a subset of systemic lupus erythematosus patients [24-26]. Recent evidence has shown that expression levels of the GEF RasGRF1 regulate constitutive MMP-9 production in human melanoma cells [27]. RasGRF1 displays *in vitro *and *in vivo *exchange activity against H-Ras [28], as well as against the Rho family GTPase Rac [29,30]. RasGRF1 activity can also be regulated by protease-dependent post-translational modification, as calpain-dependent cleavage of RasGRF1 enhances its Ras-activating capacity *in vitro *and *in vivo *[31]. Given the similarities between FLS and transformed cancer cells, we examined the expression of RasGRF1 in RA and non-RA synovial tissue and FLS, providing evidence that elevated RasGRF1 expression and post-translational modification of this protein in RA synovial tissue may contribute to joint destruction by stimulating MMP-3 production.

## Materials and methods

### Patients and synovial tissue samples

Synovial biopsy samples were obtained by arthroscopy, as previously described [32], from an actively inflamed knee or ankle joint, defined by both pain and swelling, of patients with RA (n = 10) [33], with reactive arthritis (ReA) (n = 107) [34], or with inflammatory osteoarthritis (OA) (n = 104) [35]. Patient characteristics are detailed in Table 1. All patients provided written informed consent prior to the start of the present study, which was approved by the Medical Ethics Committee of the Academic Medical Center, University of Amsterdam, The Netherlands.

**Table 1:** Clinical features of rheumatoid arthritis, reactive arthritis and osteoarthritis patients included in the study

Diagnosis	Characteristic	Median (range)
Rheumatoid arthritis	Age (years)	55 (30 to 68)
	Male:female	6:4
	Disease duration (months)	84 (2 to 360)
	Erythrocyte sedimentation rate (mm/hour)	64 (2 to 107)
	Rheumatoid factor	21 (0 to 138)
Reactive arthritis	Age (years)	33 (22 to 39)
	Male:female	4:3
	Disease duration (months)	2.5 (1 to 14)
	Erythrocyte sedimentation rate (mm/hour)	5 (0 to 14)
	Rheumatoid factor	0 (0 to 1)
Osteoarthritis	Age (years)	72.5 (54 to 83)
	Male:female	2:2
	Disease duration (months)	66 (6 to 180)
	Erythrocyte sedimentation rate (mm/hour)	9.5 (5 to 43)
	Rheumatoid factor	0 (0 to 1)

### Immunohistochemical analysis

Serial sections from at least six different biopsy samples per patient were cut with a cryostat (5 μm) and fixed with acetone, and the endogenous peroxidase activity was blocked with 0.3% hydrogen peroxide in 0.1% sodium azide/PBS. Sections were stained overnight at 4°C with mAbs against MMP-1 (MAB 1346) and against MMP-3 (MAB 1339) (both from Chemicon International, Temicula, CA, USA) and with rabbit polyclonal antibodies recognizing RasGRF1 (SC-863) (Santa Cruz Biotechnology, Santa Cruz, CA, USA), and anti-IL-6 (Department of Nephrology, Leiden University Medical Center, Leiden, The Netherlands). For control sections, primary antibodies were omitted or irrelevant immunoglobulins were applied.

Sections were then washed and incubated with goat anti-mouse horseradish peroxidase (HRP)-conjugated antibodies or swine anti-rabbit-HRP-conjugated antibodies (Dako, Glostrup, Denmark), followed by incubation with biotinylated tyramide and streptavidin–HRP, and development with amino-ethylcarbazole (Vector Laboratories, Burlingame, CA, USA) [36]. Sections were then counterstained with Mayer's hematoxylin (Perkin Elmer Life Sciences, Boston, MA, USA) and mounted in Kaiser's glycerol gelatin (Merck, Darmstadt, Germany).

### Digital image analysis

For quantitative analysis of protein expression, stained slides were randomly coded by an independent observer, blinded to antibodies used and clinical diagnosis. Stained sections were analyzed by computer-assisted image analysis using the Qwin analysis system (Leica, Cambridge, UK) as previously described in detail [37]. Values of integrated optical densities/mm^2 ^and the number of positive cells/mm^2 ^were obtained for both the intimal lining layer and the synovial sublining, and were corrected for total number of nucleated cells/mm^2^.

### Immunohistochemical double staining

To detect potential cell-specific expression of RasGRF1 in synovial tissue, tissue sections were incubated with anti-RasGRF1 antibodies overnight at 4°C, followed by serial incubation with swine anti-rabbit-HRP antibodies, biotinylated tyramine, and streptavidin–HRP. Sections were then labeled for 1 hour at room temperature with FITC-conjugated antibodies to detect T lymphocytes (anti-CD3, clone SK7; Becton Dickinson, San Jose, CA, USA), FLS (anti-CD55, mAB67; Serotec, Oxford, UK), and macrophages (anti-CD68, clone DK25; Dako), followed by incubation with alkaline phosphatase-conjugated goat anti-mouse antibody (Dako). HRP staining was developed as above, and alkaline phosphatase staining was developed using an AP Substrate III kit (SK-5300; Vector Laboratories) according to the manufacturer's instructions.

### Fibroblast-like synoviocyte culture and transfection with cDNA and locked nucleic acids

RA FLS and OA FLS were cultured as previously described [38]. FLS were used between passages 4 and 9 and were cultured in medium containing 10% FCS. To examine the influence of RasGRF1 overexpression on FLS MMP production, 2 × 10^5 ^RA FLS were plated overnight in six-well plates and were then transfected with 7.5 μg control pCDNA3 or pCDNA3 encoding full-length human RasGRF1 (provided by Dr R. Zippel, University of Milan, Milan, Italy) using Lipofectamine 2000 transfection reagent (Invitrogen, Verviers, Belgium) as per the manufacturer's instructions. Culture medium was replaced with medium containing 1.0% FCS after 24 hours, and cells were harvested 48 hours post-transfection.

RasGRF1 expression in FLS was silenced using RasGRF1-specific and control locked nucleic acids (LNA) designed with online software [39] (synthesized by Exiqon A/S, Vedbaek, Denmark). The LNA oligonucleotides used were RasGRF1 (TTGcgttaccttTGCt – LNA nucleotides in uppercase letters, DNA nucleotides in lowercase letters), and as a negative control we used a scrambled RasGRF1 sequence (GTAcagcaagatTGGg). LNA transductions were performed with Lipofectamine 2000 transfection reagent and 50 nM LNA. Culture medium was replaced with starvation medium (1% FCS in DMEM) after 24 hours and cells were harvested after an additional 24 hours.

### Protein preparation and immunoblotting

FLS were lysed in Laemli's buffer. Frozen synovial biopsies were homogenized and proteins were solubilized using a ReadyPrep™ Sequential Extraction Kit (BioRad, Hercules, CA, USA). The protein content was quantified using a BCA Protein Assay Kit (Pierce, Rockford, IL, USA). Equivalent amounts of protein were resolved by electrophoresis on NuPage 4 to 12% Bis–Tris gradient gels (Invitrogen) and were transferred to polyvinylidene difluoride membrane (BioRad). Proteins were detected by immunoblotting with anti-RasGRF1 antibodies (SC-863 and SC-224; Santa Cruz), actin antibodies (Santa Cruz) or tubulin antibodies (Sigma Aldrich, St Louis, MO, USA), followed by extensive washing, incubation with HRP-conjugated anti-rabbit or anti-mouse immunoglobulin antibodies (BioRad) and enhanced chemiluminescence detection (Pierce). For quantitative analysis of RasGRF1 expression, staining was detected using IRDye 680-labeled or 800-labeled antibodies and an Odyssey Imager (LI-COR, Bad Homburg, Germany), and was quantified using Odyssey 3.0 software.

### Measurement of MMP-1, MMP-3, TIMP-1, IL-6 and IL-8 production by fibroblast-like synoviocytes

Medium was removed from FLS 24 hours after introduction of cDNA or LNA, and was replaced with starvation medium. After 24 hours, cell-free tissue culture supernatants were harvested and analyzed using ELISA kits for MMP-1, MMP-3, TIMP-1 (all from R&D Systems Europe Ltd, Abingdon, UK), IL-6 and IL-8 (both from Sanquin Reagents, Amsterdam, The Netherlands), according to the manufacturers' instructions.

### Immunofluorescence staining

Synovial tissue sections were incubated with primary anti-RasGRF1 antibodies overnight at 4°C, followed by incubation for 30 minutes with Alexa-594-conjugated goat anti-rabbit antibodies (Molecular Probes Europe, Leiden, the Netherlands). Sections were then incubated with mouse monoclonal antibodies against MMP-1, MMP-3, or IL-6, followed by incubation with Alexa-488-conjugated goat anti-mouse antibody (Molecular Probes Europe), mounting in Vectashield (Vector Laboratories) and analysis using a fluorescence microscope (Leica DMRA) coupled to a CCD camera and Image-Pro Plus software (Media Cybernetics, Dutch Vision Components, Breda, the Netherlands).

### Statistical analysis

Wilcoxon's nonparametric signed ranks test was used to compare protein expression between the intimal lining layer and the synovial sublining layer within diagnostic groups. As no trend towards a difference in RasGRF1 expression was found between inflammatory OA and ReA synovial tissues, these two nonerosive groups were combined as non-RA samples for further analyses. The Mann–Whitney *U *test was used for the comparison of RasGRF1 expression between diagnostic groups. Correlations between RasGRF1 expression and MMP-1, MMP-3 and IL-6 expression in synovial tissue were assessed by Spearman's rank correlation coefficient. ELISA results were examined using Student's *t *test. *P *< 0.05 was considered statistically significant. There was no correction for multiple comparisons due to the exploratory nature of the study.

## Results

### Expression of RasGRF1 in RA and non-RA synovial tissue

**Figure 1:**
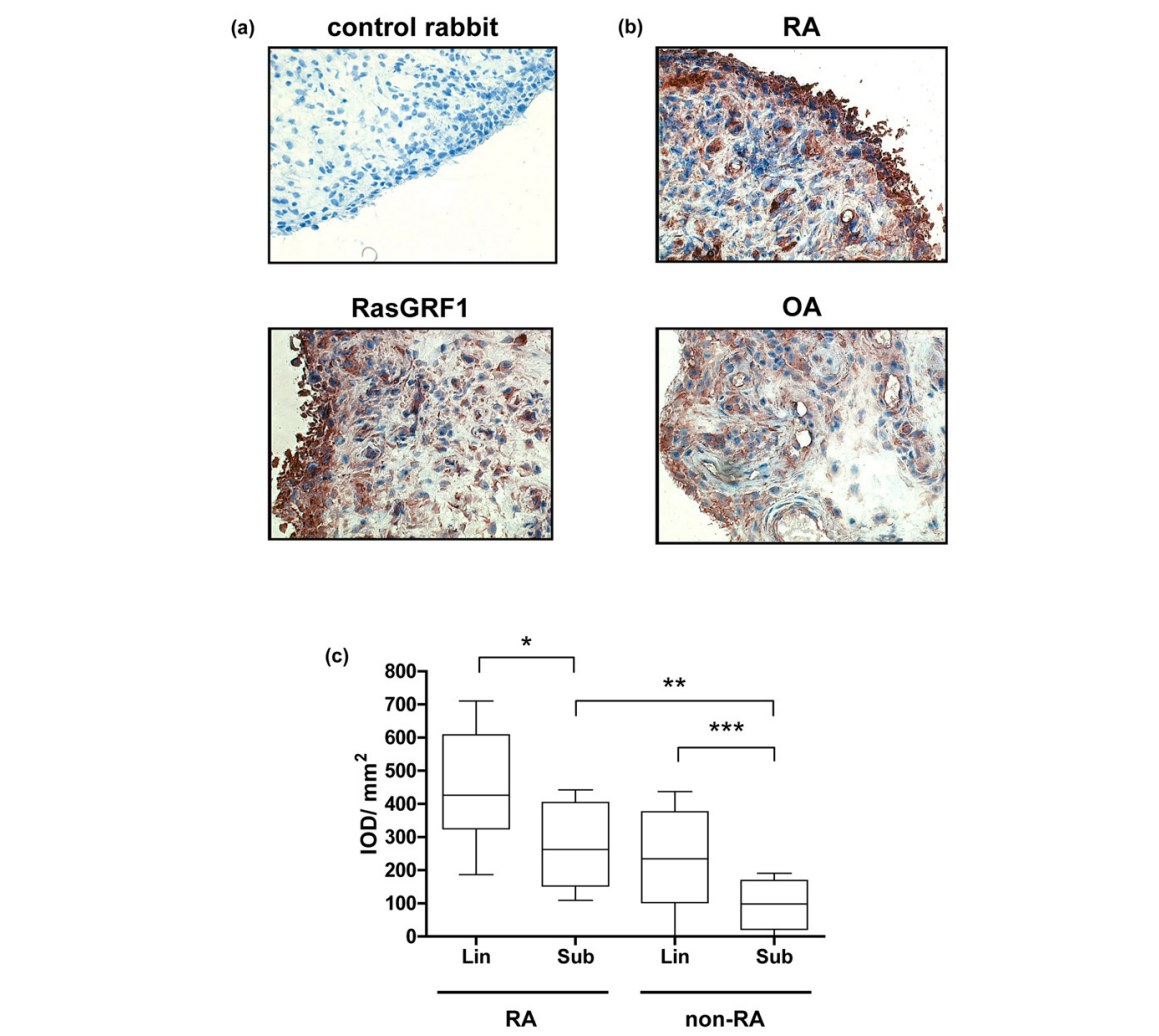
Detection of RasGRF1 protein expression in rheumatoid arthritis and non-rheumatoid arthritis synovial tissue. **(a) **Representative staining of rheumatoid arthritis (RA) synovial tissue with control and anti-Ras guanine nucleotide-releasing factor 1 (anti-RasGRF1) antibodies. **(b) **Representative staining of RA and osteoarthritis (OA) synovial tissue with anti-RasGRF1 antibodies. Staining was developed with amino-ethylcarbazole (red), and was counterstained with Mayer's hematoxylin. Magnification × 100. **(c) **Quantitative analysis of Ras signaling protein expression in RA and non-RA (OA and reactive arthritis) synovial tissue. Integrated optical densities (IOD)/mm^2^, corrected for nucleated cells, for staining of the synovial sublining (sub) and intimal lining (lin) layer of 10 RA patients and 11-non-RA (four inflammatory OA, seven reactive arthritis) patients with anti-RasGRF1 antibodies. IOD values were calculated by computer-assisted image analysis. Box plots, 25th to 75th percentiles; lines within each box, median; lines outside boxes, 10th and 90th percentiles. Bars indicate statistically significant differences in protein expression between sublining and intimal lining layer tissues within diagnostic groups and between diagnostic groups. *P < 0.05, **P < 0.01, ***P < 0.005.

To gain insight into potential involvement of RasGRF1 in RA, immunohistochemical staining was performed on RA synovial tissue using RasGRF1-specific antibodies. While no specific staining was observed with irrelevant control rabbit antibodies, robust staining was observed in RA synovial tissue with anti-RasGRF1 antibodies (Figure 1a). RasGRF1 staining was most apparent throughout the intimal lining layer, but was also observed in infiltrating mononuclear cells found in the synovial sublining.

Initial qualitative analysis of RasGRF1 expression in RA and inflammatory OA synovial tissue suggested that RasGRF1 expression was elevated in RA synovial tissue (Figure 1b). We therefore compared RasGRF1 expression in RA and non-RA (inflammatory OA and ReA) synovial tissue quantitatively, using digital image analysis (Figure 1c). Preliminary analyses indicated no differences in RasGRF1 expression between inflammatory OA and ReA synovial tissue, either in the intimal lining layer (mean integrated optical density/mm^2 ^± standard error of the mean: OA, 259.0 ± 131.6; ReA, 263.4 ± 77.0) or in the synovial sublining layer (OA, 113.3 ± 55.7; ReA, 135.6 ± 51.9) (data not shown). These two non-erosive groups were therefore combined as non-RA for further analyses. Comparing RA with non-RA synovial tissue, RasGRF1 expression was elevated in the RA (*P *< 0.05) and in the non-RA (*P *< 0.01) intimal lining layer as compared with the synovial sublining. RasGRF1 expression was enhanced in the synovial sublining of RA tissue as compared with non-RA synovial tissue (*P *< 0.01), and a trend towards enhanced RasGRF1 expression was observed in the RA intimal lining layer. Correction of RasGRF1 expression for the number of RasGRF1-positive cells confirmed that RasGRF1 expression was enhanced in both the synovial sublining (*P *< 0.005) and the intimal lining layer (*P *< 0.05) of RA patients compared with non-RA patients (data not shown).

**Figure 2:**
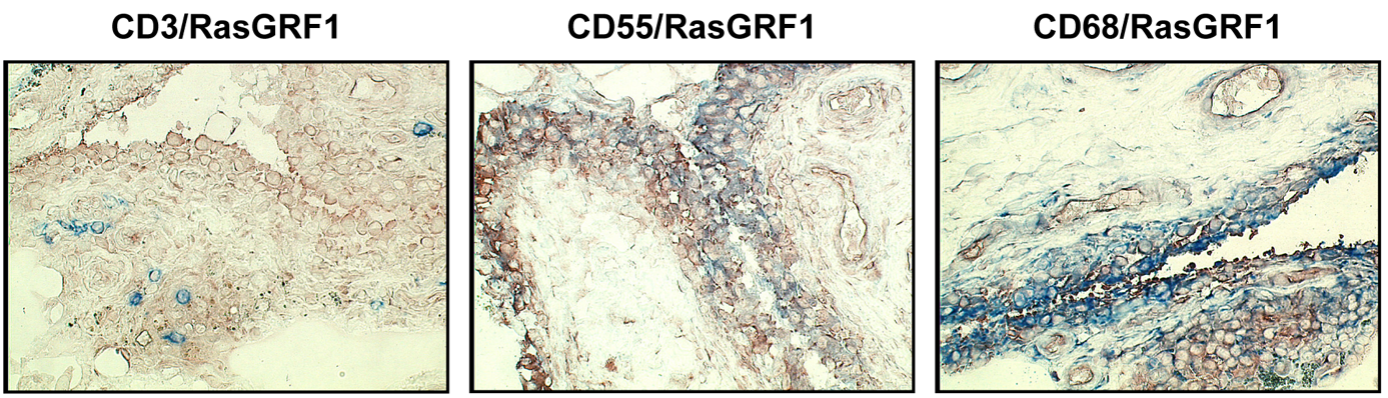
Representative double staining of rheumatoid arthritis synovial tissue with antibodies against RasGRF1 and cell-specific markers. Synovial tissue sections were stained overnight with antibodies against Ras guanine nucleotide-releasing factor 1(RasGRF1), followed by antibodies against CD3, CD55, and CD68. After biotin tyramide enhancement, staining was developed with amino-ethylcarbazole (red, RasGRF1) and Fast blue (blue, cell-specific markers). Magnification × 100.

Qualitative double-labeling of RA synovial tissue with antibodies recognizing RasGRF1 and markers for T lymphocytes (CD3), FLS (CD55), and macrophages (CD68) revealed that RasGRF1 expression was restricted to FLS and macrophages (Figure 2).

### RasGRF1 expression in RA and non-RA fibroblast-like synoviocytes

To independently confirm RasGRF1 expression in synovial tissue and FLS detected by immunohistochemistry, we performed immunoblotting experiments on lysates derived from intact RA and OA synovial biopsies, and from RA and OA FLS.

**Figure 3:**
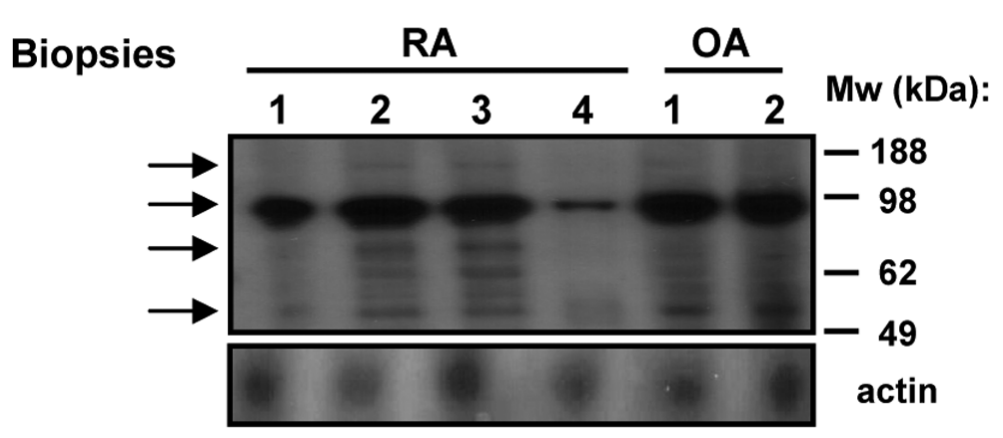
RasGRF1 is expressed as a truncated protein in synovial tissue.  Immunoblot analysis of Ras guanine nucleotide-releasing factor 1 (RasGRF1) and actin in rheumatoid arthritis (RA) and osteoarthritis (OA) synovial biopsy lysates. The 98 kDa, 75 kDa and 54 kDa proteins reacting with RasGRF1 antibodies, and the expected position of full-length 140 kDa RasGRF1, are indicated on the left by arrowheads. Relative mobility of molecular weight (Mw) standards (kDa) indicated to the right.

In protein lysates derived from intact RA and OA synovial biopsies (Figure 3), we were unable to detect full-length 140 kDa RasGRF1. We did, however, observe prominent expression of a 98 kDa truncation product, and lower and variable levels of 75 and 54 kDa truncation products. These C-terminal fragments are thought to be generated by calpain-dependent cleavage, resulting in constitutive activation of RasGRF1 [27,31].

**Figure 4:**
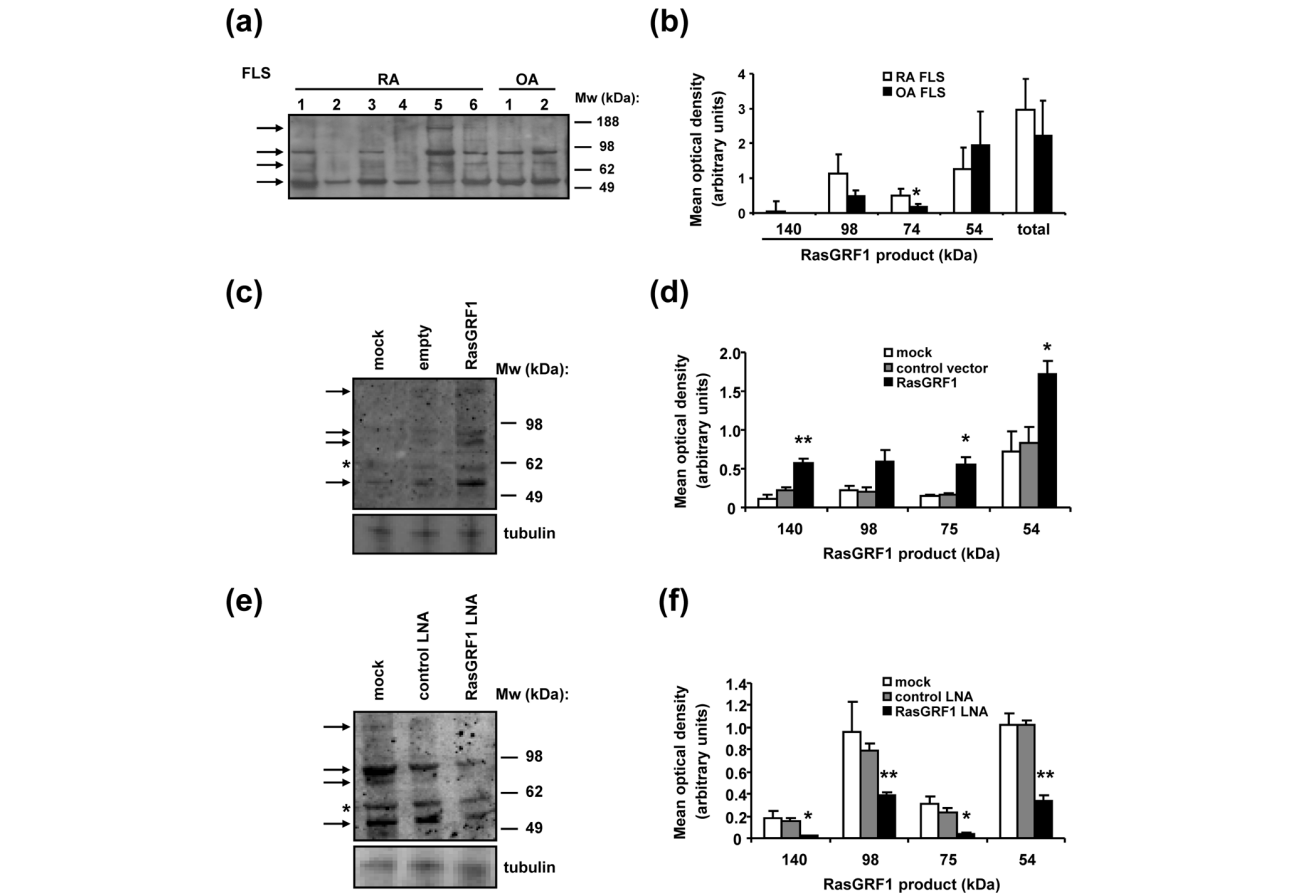
RasGRF1 is expressed as a truncated protein in fibroblast-like synoviocytes.  **(a) **Immunoblot analysis of Ras guanine nucleotide-releasing factor 1 (RasGRF1) in rheumatoid arthritis (RA) and osteoarthritis (OA) fibroblast-like synoviocytes (FLS). The 140 kDa, 98 kDa, 75 kDa and 54 kDa proteins reacting with RasGRF1 antibodies are indicated on the left by arrowheads. Relative mobility of molecular weight (Mw) standards (kDa) indicated to the right. **(b) **Expression of 140 kDa, 98 kDa, 75 kDa, and 54 kDa RasGRF1 polypeptides as well as the total RasGRF1 signal, normalized to tubulin expression, was quantified in RA (n = 5) and OA (n = 5) FLS lines, and expressed as mean optical density ± standard error of the mean (SEM). **(c) **Overexpression of RasGRF1 in RA FLS. RA FLS were treated with transfection reagent alone (mock) or transfected with empty (control) vector or vector encoding RasGRF1, and cell lysates immunoblotted with antibodies against RasGRF1 (upper panel) and tubulin (lower panel). Expression of full-length and truncated RasGRF1 polypeptides is indicated with arrows, and a 60 kDa polypeptide with an asterisk. **(d) **Expression of 140 kDa, 98 kDa, 75 kDa, and 54 kDa RasGRF1 polypeptides following transfection of RA FLS with empty vector or RasGRF1, normalized to tubulin expression was quantified and expressed as mean optical density ± SEM (middle panel) (n = 4). **(e) **Silencing of RasGRF1 expression with locked nucleic acid (LNA). RA FLS were treated with transfection reagent alone (mock) or transduced with control or RasGRF1 LNA and lysates assessed for expression of RasGRF1 (upper panel) and tubulin (lower panel) by immunoblotting. **(f) **Quantitative analysis of (e) as in (d). *P < 0.05, **P < 0.01 compared with controls.

In analyses of FLS lysates, full-length 140 kDa RasGRF1 was detected by immunoblotting in only one of six RA FLS lines (RA FLS5), and in neither of two OA FLS lines tested (Figure 4a). In contrast, a 54 kDa RasGRF1 C-terminal fragment was detected in all RA and OA FLS lines, a 75 kDa fragment in three of five RA FLS lines and in both OA FLS lines, and a 98 kDa C-terminal fragment in four of six RA lines and in both OA lines. Quantitative analysis of RasGRF1 protein expression in five RA lines and five OA FLS lines revealed no significant difference in total RasGRF1 expression (Figure 4b). With the exception of the 74 kDa RasGRF1 fragment, which was detected at lower levels in RA FLS (*P *< 0.05), the other RasGRF1 truncation fragments, as well as full-length RasGRF1, were expressed at similar levels in RA FLS and OA FLS.

To verify that the observed truncation products were derived from RasGRF1, rather than from nonspecific interactions with the antibodies, we performed additional experiments. First, RA FLS were transfected with cDNA encoding full-length RasGRF1 (Figure 4c, d). Quantitative analysis of proteins detected by immunoblotting demonstrated that transfection of RA FLS with RasGRF1 cDNA encoding full-length RasGRF1 resulted in the enhanced expression of the 140 kDa (*P *< 0.01), 98 kDa and 75 kDa (*P *< 0.05), and 54 kDa (*P *< 0.05) forms of RasGRF1. Second, we silenced RasGRF1 expression by transduction of RA FLS with RasGRF1-specific LNA. LNA are antisense nucleotide analogs containing methylene bridges that mimic the RNA monomer structure, and disrupt gene expression by promoting mRNA degradation and/or preventing gene product translation [40]. RasGRF1-specific LNA decreased RasGRF1 expression in RA FLS compared with control scrambled LNA (Figure 4e), while leaving tubulin expression unaffected. Significant decreases in the expression of full-length 140 kDa RasGRF1 (*P *< 0.05) and of the 98 kDa (*P *< 0.01), 75 kDa (*P *< 0.05) and 54 kDa (*P *< 0.01) forms were achieved (Figure 4f). Exposure of FLS to transfection reagent alone resulted in the generation of an additional 60 kDa polypeptide (mock-treated FLS in Figures 4c and 4e, asterisk) not observed in synovial biopsies or untreated FLS, possibly due to activation of an unidentified cellular protease.

### Effects of changes in RasGRF1 expression on RA fibroblast-like synoviocyte MMP-3 production *in vitro*

**Figure 5:**
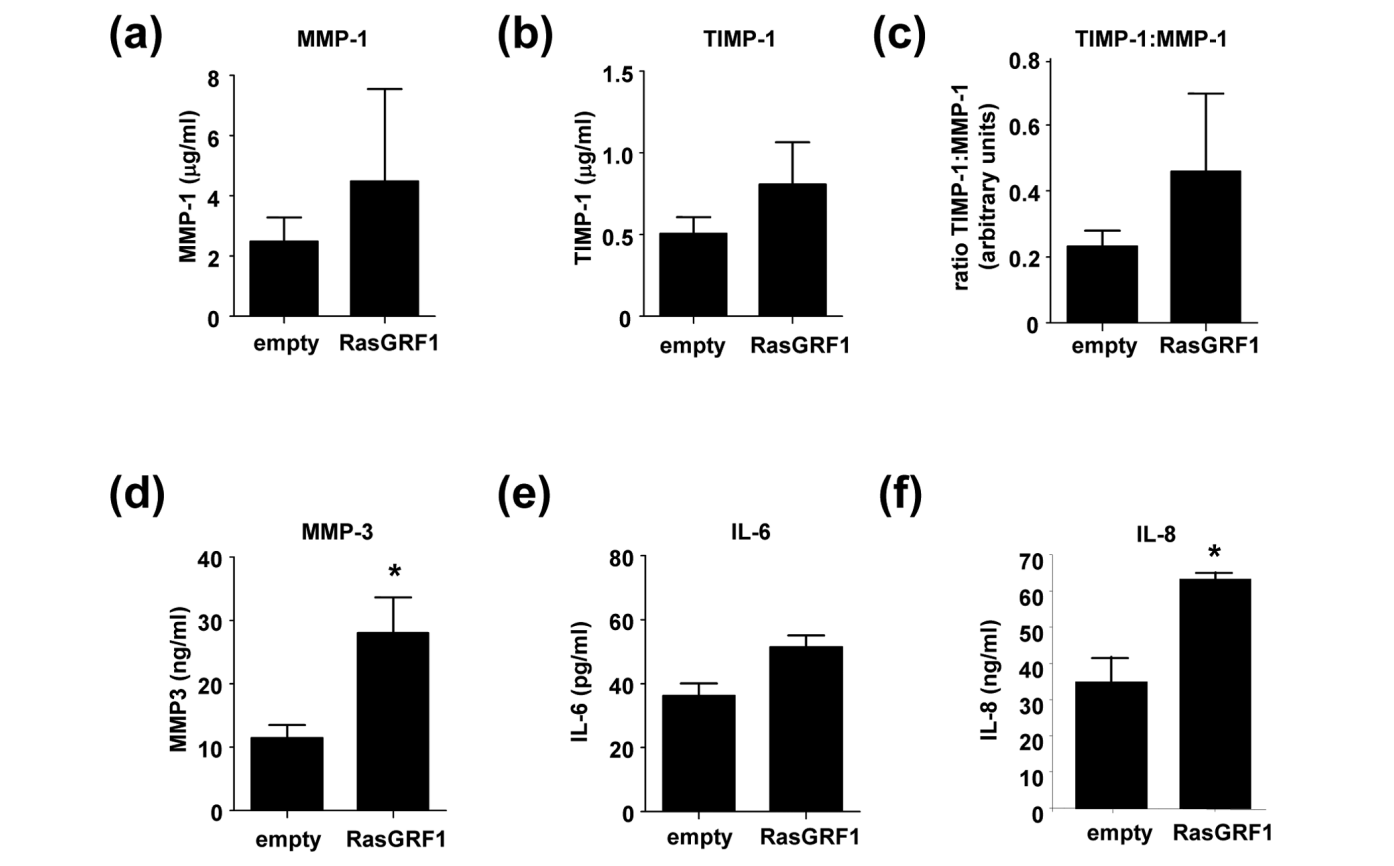
Effect of RasGRF1 overexpression on rheumatoid arthritis fibroblast-like synoviocyte matrix metalloproteinase and cytokine production. Tissue culture supernatants from rheumatoid arthritis fibroblast-like synoviocytes transfected with empty vector or with Ras guanine nucleotide-releasing factor 1 (RasGRF1) were harvested and assessed for production of **(a) **matrix metalloproteinase (MMP)-1, **(b) **TIMP-1, **(c) **the ratio of TIMP-1 to MMP-1, **(d) **MMP-3, **(e) **IL-6 (n = 4 each) and **(f) **IL-8 (n = 3) by ELISA. *P < 0.05 compared with controls.

As RasGRF1 expression levels regulate MMP production in cancer cell lines [27], we examined whether modulation of RasGRF1 expression in RA FLS might also regulate constitutive MMP and cytokine production. Quantitative analysis of FLS tissue culture supernatants demonstrated that RasGRF1 overexpression had no effect on FLS production of MMP-1 (Figure 5a) or of TIMP-1 (Figure 5b). Additionally, the ratio of TIMP-1 expression relative to MMP-1 was unaffected (Figure 5c). Forced expression of RasGRF1, however, induced an approximately 150% increase in MMP-3 production (mean ± standard error of the mean, 27.99 ± 5.62 ng/ml) compared with FLS transfected with empty control vector alone (11.47 ± 2.02 ng/ml) (*P *< 0.05) (Figure 5d). Enhancing RasGRF1 expression had no effect on spontaneous IL-6 production by RA FLS (Figure 5e), but did increase spontaneous IL-8 secretion by approximately twofold (*P *< 0.05) (Figure 5f).

**Figure 6:**
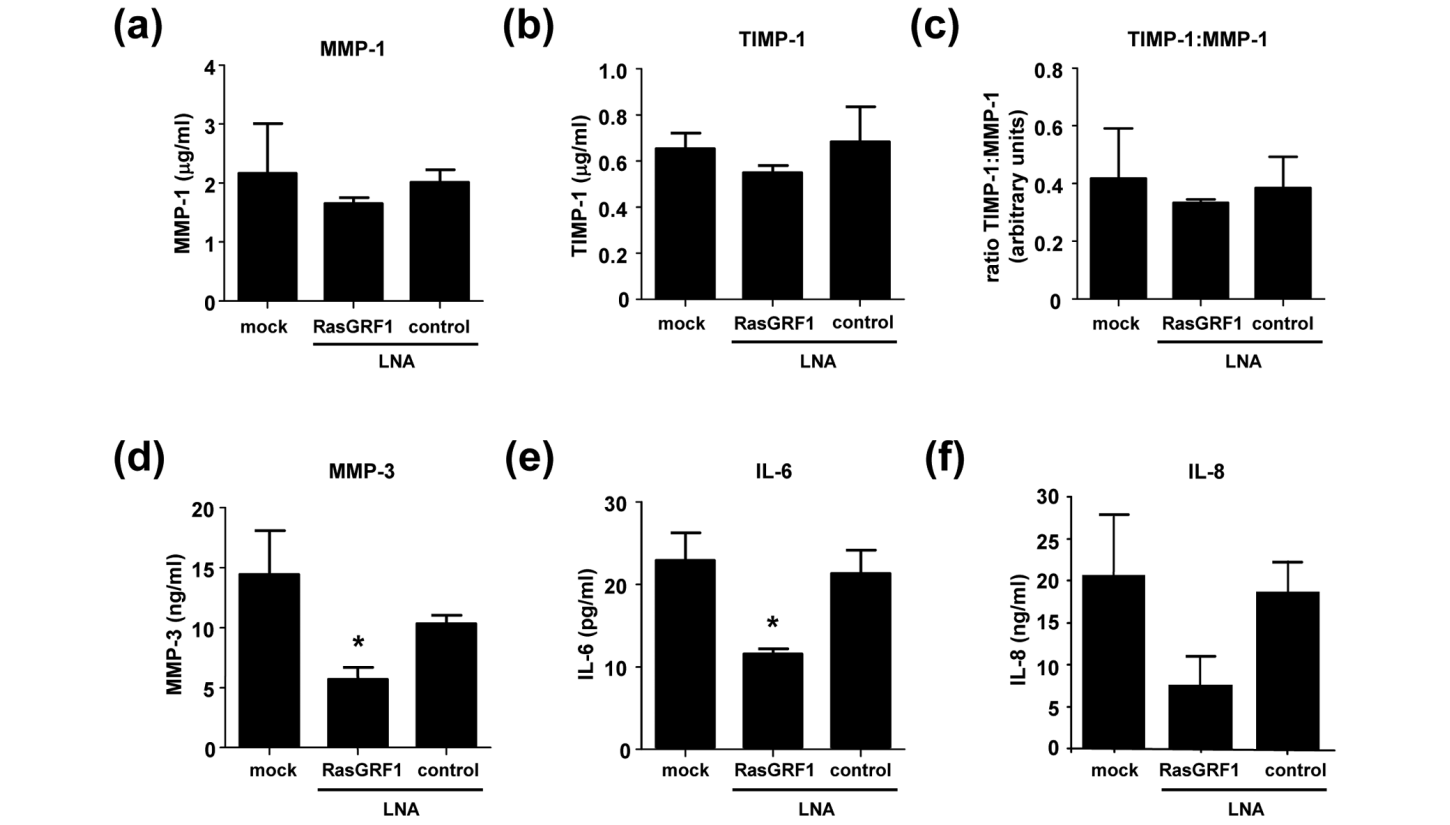
Effect of RasGRF1 gene silencing on rheumatoid arthritis fibroblast-like synoviocyte matrix metalloproteinase and cytokine production.  Tissue culture supernatants from rheumatoid arthritis fibroblast-like synoviocytes treated with transfection reagent alone (mock) or transfected with control or Ras guanine nucleotide-releasing factor 1 (RasGRF1) locked nucleic acid (LNA) were harvested and assessed for production of **(a) **matrix metalloproteinase (MMP)-1, **(b) **TIMP-1, **(c) **the ratio of TIMP-1 to MMP-1, **(d) **MMP-3, **(e) **IL-6 (n = 4 each) and **(f) **IL-8 (n = 3) by ELISA. *P < 0.05 compared with controls.

To determine whether RasGRF1 was required for spontaneous MMP or cytokine production, we silenced RasGRF1 gene expression using LNA. Again, modulation of RasGRF1 expression failed to influence MMP-1 and TIMP-1 production, or the ratio of TIMP-1 relative to MMP-1 (Figure 6a to 6c). A significant suppression of spontaneous MMP-3 production was observed in tissue culture supernatants of FLS transduced with RasGRF1-specific LNA (Figure 6d) (*P *< 0.05), as compared with FLS treated with transfection reagent alone or in combination with control scrambled LNA. Although overexpression of RasGRF1 in RA FLS failed to enhance basal IL-6 production (Figure 5e), IL-6 levels were significantly decreased following silencing of RasGRF1 expression (Figure 6e) (*P *< 0.05). An apparent 67% reduction in spontaneous IL-8 production was also noted, but this did not reach statistical significance (*P *= 0.069) (Figure 6f).

### Relationship between RasGRF1 expression and matrix metalloproteinase production in RA synovial tissue

**Figure 7:**
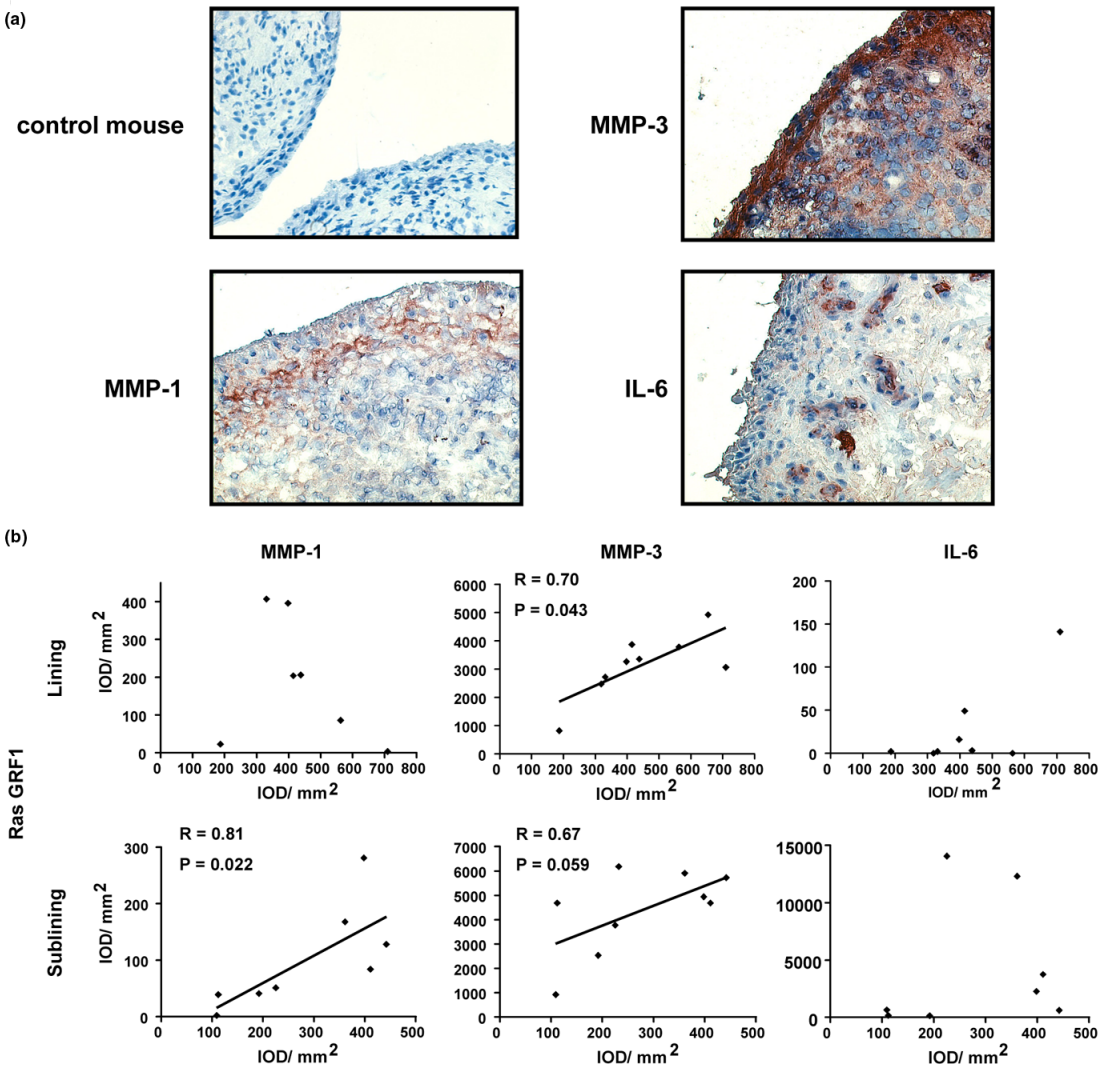
Association of RasGRF1 expression with matrix metalloproteinase production in rheumatoid arthritis synovial tissue. **(a) **Representative staining of rheumatoid arthritis synovial tissue with control and anti-matrix metalloproteinase (MMP)-1, MMP-3, and IL-6 antibodies (magnification × 100). **(b) **Correlation of Ras signaling protein expression with MMP-1 and MMP-3 production in RA synovial tissue. Pearson *R *values and *P *values are indicated. IOD, integrated optical density; RasGRF1, Ras guanine nucleotide-releasing factor 1.

Our *in vitro *data indicated an important role for RasGRF1 in regulating MMP-3 expression in RA FLS. We therefore examined whether expression of RasGRF1 was associated with MMP-3 production in RA synovial tissue. Immunohistochemical analysis demonstrated that MMP-1, MMP-3, and IL-6 were readily detected in RA synovial tissue (Figure 7a). RasGRF1 expression demonstrated a strong positive correlation (*R *= 0.81, *P *= 0.022) with MMP-1 in the RA synovial sublining, but not in the intimal lining layer (Figure 7b). Instead, a positive correlation between RasGRF1 and MMP-3 expression was observed in the intimal lining layer (*R *= 0.70, *P *= 0.043). In non-RA patients, no significant association between RasGRF1 and MMP-1 (synovial sublining: *R *= 0.17, *P *= 0.703; intimal lining layer: *R *= -0.89, *P *= 0.083) or MMP-3 (synovial sublining: *R *= 0.83, *P *= 0.058; intimal lining layer: *R *= -0.20, *P *= 0.917) expression was observed (data not shown). No correlation was observed between RasGRF1 expression and IL-6 expression in either RA or non-RA patient cohorts (Figure 7b and data not shown).

**Figure 8:**
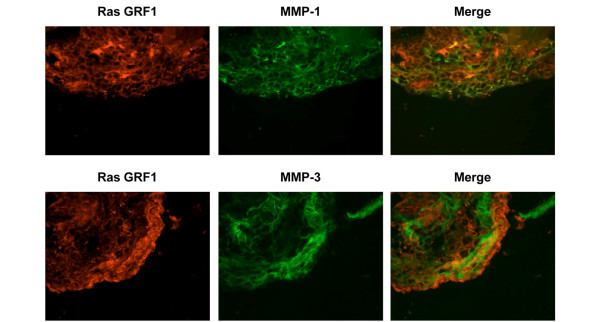
Double immunofluorescence labeling of RasGRF1, MMP-1 and MMP-3 in rheumatoid arthritis synovial tissue. Rheumatoid arthritis synovial tissue was stained with combinations of anti-Ras guanine nucleotide-releasing factor 1 (anti-RasGRF1) and either anti-matrix metalloproteinase (MMP)-1 (upper panels) or anti-MMP-3 (lower panels). Sections were then stained with fluorochrome-conjugated anti-rabbit immunoglobulin (red) and anti-mouse IgG (green) antibodies to visualize RasGRF1 and MMP expression, respectively. Colocalization of RasGRF1 with MMPs is visualized by yellow staining in merged images (right panels).

Double immunofluorescent staining revealed colocalization of RasGRF1 with MMP-1 and MMP-3 in RA synovial tissue (Figure 8). Colocalization of RasGRF1 with MMP-1 was observed in the synovial sublining (Figure 8, upper panels), while RasGRF1 colocalization with MMP-3 was restricted to the intimal lining layer (Figure 8, lower panels). Together, these data indicate that RasGRF1 may contribute to RA FLS MMP-3 production *in vivo*.

## Discussion

Our results demonstrate that RasGRF1 regulates spontaneous MMP-3 production in RA FLS, and suggest that overexpression of RasGRF1 sensitizes signaling pathways promoting MMP-3 production and joint destruction in RA. RasGRF1 specifically activates H-Ras, but not other Ras homologs *in vivo *[28], and RasGRF1 activation of H-Ras induces constitutive MMP-9 production in human melanoma cells [27]. RasGRF1 can also activate the Rho family GTPase Rac1 [29,30], and a role for Rac1 – potentially via activation of JNK – has been recently shown in the regulation of RA FLS proliferation and invasiveness [41]. Data have been reported indicating that RasGRF1 can also stimulate GTP exchange on R-Ras *in vitro*, although this GEF activity has yet to be verified *in vivo *[42,43].

Our data raise the possibility that changes in the expression of GEFs, such as RasGRF1, or of negatively regulatory GAPs may be more relevant to the pathology of RA than GTPase expression levels. We observe a strong positive correlation between RasGRF1 expression in RA synovial tissue on the one hand, and production of MMP-1 and MMP-3 on the other. Such an association is not clearly observed in non-RA synovial tissue. Consistent with the notion that RasGRF1 is involved in the regulation of MMPs, we find that RasGRF1 expression colocalizes to synovial cells producing MMP-1 and MMP-3 *in situ*, and that modulation of RasGRF1 in RA FLS *in vitro *regulates spontaneous MMP-3 production by these cells. The inability of RasGRF1 modulation to regulate MMP-1 production in RA FLS, despite the positive association of expression of these proteins in the synovial sublining *in vivo*, may indicate that other RasGRF1-expressing cells – namely, macrophages – are a more important source of MMP-1 *in vivo*. Consistent with this, we observe a relationship between RasGRF1 and MMP-1 in the synovial sublining, where macrophages constitute the predominant cell population. Additionally, co-localization of cells expressing RasGRF1 and MMP-1 is most apparent in the synovial sublining layer. Further direct studies will be needed to examine whether RasGRF1 regulates MMP-1 production in synovial macrophages. Alternatively, RasGRF1-dependent secretion of IL-8 or other as yet unidentified inflammatory cytokines may indirectly promote MMP-1 production *in vivo *through the recruitment and/or activation of leukocytes.

We provide additional *in vitro *evidence that although many FLS stimuli regulate both MMP-1 and MMP-3 expression, regulation of these two proteases is not requisitely coupled. For instance, adhesion of RA FLS to laminin-111 in the presence of tumor growth factor beta induces expression of MMP-3 but not of MMP-1 [44]. Inhibition of JNK can partially block TNFα-induced MMP-1 production by RA FLS, but MMP-3 production is independent of JNK [45]. Reciprocally, mitogen-activated protein kinase-activated protein kinase 2 (MK2) regulates MMP-3 secretion, but not MMP-1, in OA chondrocytes [46]. The fact that regulation of MMP-1 is uncoupled from that of MMP-3 probably reflects differential utilization of NF-κB, activator protein-1 (AP-1), E26 transforming sequence (Ets), and hypoxia-inducible factor-1α transcription factors by the promoters of the MMP-1 and MMP-3 genes [42,47,48]. Similarly, we find that RasGRF1 is necessary for spontaneous IL-6 production by RA FLS, but overexpression of RasGRF1 is not sufficient to augment IL-6 secretion. This may reflect a necessary coordination of RasGRF1 signaling with other signaling pathways, such as previously reported cooperative effects between Ras GTPase and c-*myc *pathways in the regulation of RA FLS activation [22]. Further definition of pathways by which RasGRF1 modulates MMP and cytokine production will require identification of the immediate downstream target(s) of this GEF in FLS.

While RasGRF1 expression is sufficient and required for spontaneous MMP-3 production in RA FLS, similar effects of RasGRF1 on MMP-1, TIMP-1 and IL-6 are not observed. Stimuli that activate RasGRF1 include ligands for both tyrosine kinase receptors and G-protein-coupled receptor [49]. Examples of receptors known to regulate RasGRF1 and expressed in RA synovial tissue include those for lysophosphatidic acid and muscarinic acid, N-methyl-D-aspartic acid, and nerve growth factor [50-53]. In preliminary studies, we have found that silencing of RasGRF1 in RA FLS has no effect on TNFα-induced or IL-1β-induced MMP-3 production (data not shown). RasGRF1 activity can also be regulated by post-translational modification, as calpain-dependent cleavage of RasGRF1 enhances Ras-activating capacity *in vitro *and *in vivo *[27,31]. Enhanced expression of RasGRF1 in RA tissue compared with non-RA tissue may sensitize RA FLS to produce MMPs in response to extracellular stimuli. This would result from disease-specific extracellular stimuli activating full-length RasGRF1, as well as constitutive signaling from post-translationally modified RasGRF1, such as the predominantly expressed 96 kDa carboxy-terminal fragment we observe in synovial tissue. Identification of the protease(s) responsible for RasGRF1 cleavage *in vivo *may lead to new therapeutic strategies in the treatment of arthritis.

## Conclusions

RasGRF1 expression and post-translational modifications regulate spontaneous MMP-3 production in RA FLS, and are associated with MMP-3 production in RA synovial tissue. Contributions of RasGRF1 to MMP-3 production in RA and other forms of arthritis will probably depend on RasGRF1 expression levels, on the extent of activating post-translational modifications of RasGRF1, and on the strength of extracellular stimuli leading to activation of residual full-length RasGRF1. Our data suggest a molecular mechanism by which Ras signaling pathways might contribute to the semi-transformed and invasive phenotype of RA FLS in the absence of oncogenic mutations in Ras superfamily GTPases.

## Abbreviations

AP-1: activator protein-1; DMEM: Dulbecco's modified Eagle's medium; ELISA: enzyme-linked immunosorbent assay; Ets: E26 transforming sequence; FCS: fetal calf serum; FLS: fibroblast-like synoviocyte; GEF: guanine nucleotide exchange factor; HRP: horseradish peroxidase; IL: interleukin; JNK: c-jun N-terminal kinase; kDa: kilodalton; LNA: locked nucleic acid; mAb: monoclonal antibody; MMP: matrix metalloproteinase; NF: nuclear factor; OA: osteoarthritis; PBS: phosphate-buffered saline; RA: rheumatoid arthritis; RasGRF1: Ras guanine nucleotide-releasing factor 1; ReA: reactive arthritis; TIMP-1: tissue inhibitor of metalloproteinases 1.

## Competing interests

The authors declare that they have no competing interests.

## Authors' contributions

JRFA performed and evaluated the immunohistochemical experiments, and contributed to the drafting of the manuscript. DdL performed the *in vitro *experiments and contributed to the drafting of the manuscript. MES and AMG also contributed to the *in vitro *experiments. MGvdS provided patient material and assembled and evaluated patient clinical data. PPT contributed to the study design, evaluation of data and drafting of the manuscript. KAR conceived the study, and contributed to the evaluation of the data and drafting of the manuscript. All authors read and approved the final manuscript.
